# Vitamin D deficiency is a risk factor for obesity and diabetes type 2 in women at late reproductive age

**DOI:** 10.18632/aging.100582

**Published:** 2013-07-22

**Authors:** EN Grineva, T Karonova, E Micheeva, O Belyaeva, IL Nikitina

**Affiliations:** ^1^ Almazov's Centre of Heart, Blood and Endocrinology, Petersburg, 197134, Russia; ^2^ I. P. Pavlov St Petersburg State Medical University, St. Petersburg, 197022, Russia

**Keywords:** Vitamin D deficiency, Diabetes Mellitus type2, obesity, overweight

## Abstract

It was suggested that glucose metabolism and body fat content depend on serum levels of 25-hydroxyvitamin D [25(OH)D]. We studied 320 healthy women at late reproductive age of 40 to 52 years old (mean age 46.1±4.5) from St. Petersburg (North-West region of Russia). 25(OH)D levels were from 19.4 to 134.0 nMol/L (mean 52.9±22.7). Vitamin D deficiency (lower than 50 nMol/L) and insufficiency (50-75 nMol/L) was revealed in 59.1% and 27.8% of women, respectively. The study showed that low 25(OH)D levels were associated with obesity (r=-0.35, p<0.01), increased plasma glucose levels after OGTT (r=-0.31, p<0.01) and decreased insulin sensitivity index (r=-0.28, p<0.01). We found that 25(OH)D levels below 50 nMol/L were associated with obesity risk (OR 2.25[1.05-3.95], CI 95%) but not with risk of impaired glucose metabolism (1.07[0.54-2.12],CI95%). Our results showed that vitamin D insufficiency is highly prevalent in the population of healthy women. Low 25(OH)D levels correlated with high body fat, glucose levels and decreased insulin sensitivity. We conclude that vitamin D deficiency is a potential risk factor for obesity and development of insulin resistance leading to diabetes type 2.

## INTRODUCTION

Approximately 1 billion people worldwide suffer from vitamin D deficiency [1-4], which may result from limited exposure to sunlight, long-term wearing of covering clothes, use of sunscreen, age as well as low consumption of food containing ergocalciferol, and malabsorption syndrome [5-6]. The vitamin D receptors (VDR) and the 1α-hydroxylase enzyme, which catalyzes the conversion of calcidiol [25-hydroxyvitamin D, 25(OH)D] to calcitriol [1,25-dihydroxyvitamin D, 1,25(OH)_2_D], were found in more than 40 human cell types [1-8], indicating its potential role in the regulation of numerous metabolic processes. According to recent data, there may be a connection between vitamin D levels and cardiometabolic diseases: obesity; impaired glucose tolerance and diabetes mellitus type 2; arterial hypertension; and atherogenic dyslipidemia. Although the mechanisms are still unclear, vitamin D deficiency is associated with a greater risk of these pathological conditions [4,6,10-14]. Furthermore, an increased body fat and obesity is associated with low *circulating* 25(OH)D level [9,14-19].

Numerous studies investigated the relationship between 25(OH)D and insulin levels. *Vitamin D receptors* found in pancreatic ß-cells launched studies on the possible effects of calcitriol on regulation of insulin production [14,15]. It is well known that Vitamin D treatment of animals with induced diabetes mellitus type 1 slows the progression of diabetes, and that high doses of vitamin D in food consumed by risk-group children are able to reduce the incidence of diabetes [20-23]. In addition, while assessing carbohydrate metabolism, it was determined that the lack of vitamin D may cause a greater level of glycemia and a higher risk of diabetes mellitus type 2 [2,4,5,14,15,22,24]. There is a link between 25(OH)D levels and insulin responsiveness of tissues as well as between glucose levels and glycosylated hemoglobin in people without diabetes mellitus type 2 [15,21]. However, data from other authors controvert the relation of vitamin D deficiency and metabolic syndrome factors [20,25]. Given these contradicting data, we sought to determine whether serum 25(OH)D concentration in late reproductive age healthy women is associated with body composition and glucose metabolism.

## RESULTS

The mean age of women followed up was 46.1±4.5 years (from 40 to 52), BMI - 30.2±6.1 kg/m^2^ (from 21.2 to 53.1). Depending on their BMI the participants were divided into groups of normal weight, overweight, and obese. Distribution between the groups was done according to waist circumference values: over or equal to 80 cm or less than 80 cm, as recommended by International Diabetes Federation [26].

The results of the study showed that 78% of women were overweight or obese, with WC more than 80 cm in 83.6%. DEXA confirmed increased fat mass in 87.7% of study population. ROC-analysis showed correlation between BMI and FMI (rang correlation coefficient R_γ_=+0.98).

Vitamin 25(OH)D serumconcentration varied from 19.4 to 134.0 nmol/L and on average was 52.9±22.7 nmol/L, at the same time in 86.9% women it was insufficient or deficient. Only 13.1% had normal levels of calcidiol. Levels of 25(OH)D in various seasons (September-November, December-February and March-May) were not significantly different. Mean calcidiol level for each season were 53.5±4.5 nmol/L, 55.2±2.4 nmol/L and 60.8±4.4 nmol/L correspondingly. Correlation analysis showed that obese women had lower 25(OH)D level than women with normal BMI (r=-0.35, p<0.01) (Table 1). We found that among subjects with serum 25(OH)D level up to 25 and 50 nmol/L obesity risk, in particular Class II and III, was higher than in subjects with 25(OH)D level >75 nmol/L.

**Table 1:** Characteristics of study population by Vitamin D status

Parametrs	Normal 25(OH)D n=42 1	25(OH)D Insufficiency n=89 2	25(OH)D Deficiency n=189 3	p (1-3)
Age, years	42.9±0,5	44.3±0,4	43.2±1.2	>0.05
Weight, kg	72.7±1.2	75.2±1.1	77.7±2.8	<0.05
BMI, kg/m^2^	27.8±0.4	28.0±0.4	28.5±1.0	<0.05
FMI, kg/m^2^	12.1±0.4	12.7±0.4	13.2±1.3	>0.05
WC, cm	81.4±1.3	88.1±0.9	90.9±2.6	<0.05
Total Fat, kg	32.8±1.3	34.3±1.2	36.2±3.3	>0.05
Total body (%Fat)	41.1±0.7	42.9±0.7	44.7±0.6	<0.05
Trunk %Fat	42.8±0.8	44.1±0.9	44.1±0.7	>0.05
Serum 25(OH)D , nMol/L	96.7±3.1	60.6±1.0	39.3±0.8	<0.01
Fasting plasma glucose, mMol/L	5.6±0.1	6.1±0.2	6.2±0.2	<0.01
Serum Insulin, IU/mL	10.7±1.7	10.3±0.9	11.6±0.8	>0.05
Plasma glucose - 120 min OGTT, mMol/L	6.9±0.5	7.4±0.2	7.6±0.2	<0.01
Serum Insulin - 120 min OGTT, IU/mL	20.5±5.5	34.7±4.0	49.7±5.8	<0.01
HOMA-IR	3.1±0.08	3.3±0.10	3.3±0.08	<0.05
HOMA-B	95.4±11.7	88.7±7.7	101.3±8.2	<0.01
ISI-(0,120)	10.0±1.0	7.9±0.5	7.4±0.4	<0.01
iPTH, pg/mL	42.0± 1.2	42.3±2.1	44.1±1.9	>0.05

Notes. BMI, body mass index; FMI, fat mass index; WC, waist circumferences; HOMA-IR, Homeostasis model assessment estimates of IR; HOMA-B, Homeostasis model assessment estimates of β-cells function; ISI-(0,120), Insulin sensitivity index; iPTH, intact parathyroid hormone; values presented are means ±S.E.M.

Serum intact parathyroid hormone (iPTH) level was normal in total study population (mean 42.0±1.2 pg/mL) and women with obesity had a tendency of negative association between iPTH and 25(OH)D levels (r=-0.2, p=0.08). Correlation analysis showed interlinks between iPTH and WC (r=0.31, p<0.05) as well as FMI (r=0.34, p<0.05).

**Figure 1:**
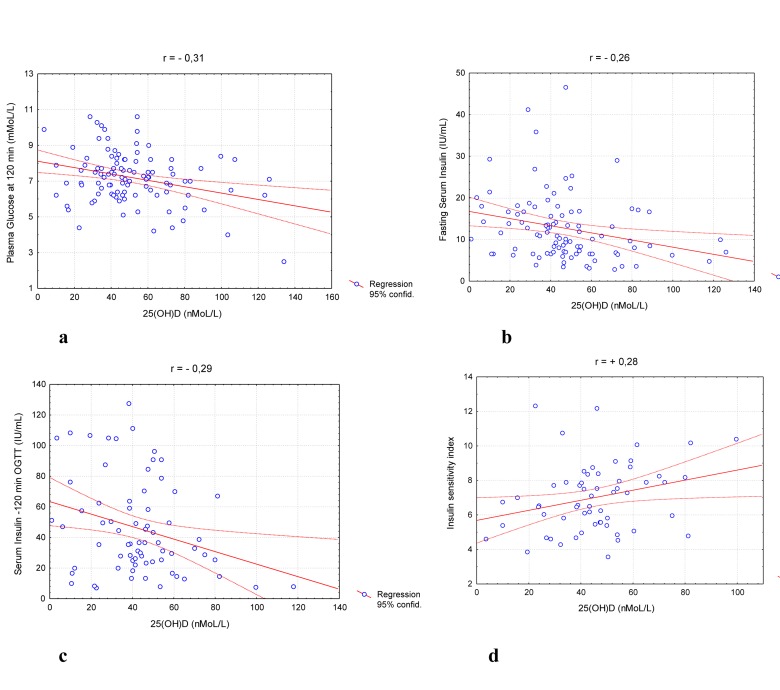
Distribution stimulated glucose (**a**), fasting (**b**) and stimulated (**c**) Insulin levels and ISI (0,120) parameters (**d**) in overweight/obese population.

The results of fasting glucose and OGTT showed diabetes mellitus type 2 in 4.3% of the participants, impaired glucose tolerance or high level of fasting glucose in 28.6% , and normal glycemia values for 67.1% of the patients. We did not find correlation between glucose level and 25(OH)D in total population. However, in overweight and obese subpopulation there were a significant correlation between fasting insulin (r=-0.26, p<0.01), 2h OGTT glucose and insulin levels (r=-0.31, p<0.01 and r=-0.29, p<0.01 accordingly) and serum 25(OH)D concentration (Figure 1.). Moreover, calcidiol levels in women with normal weight was inversely proportional to HOMA-B values (r=-0.48, p<0.01), and for those with overweight and obesity they were directly proportional to insulin sensitivity index (r=0.28, p<0.01). Study results showed that low 25(OH)D level was not significantly associated with increased risk of impaired glucose tolerance and diabetes type 2 (OR 1.07 (Table 2).

**Table 2:** Risk of metabolic diseases (OR, CI95%) in women with different vitamin D status

	25(OH)D Level <25 nMol/L	25(OH)D Level <50 nMol/L	25(OH)D Level <75 nMol/L
Obesity	1.87[0.91-3.84]	2.25[1.05-3.95]	1.86[0.86-3.95]
Obesity Class II and III	3.05[1.10-5.39]	2.15[0.69-6.64]	1.59[0.53-4.85]
WC >80 cm	2.28[1.17-4.46]	2.23[1.15-4.30]	1.87[0.99-3.54]
IGT	0.71[0.29-1.75]	0.99[0.48-2.02]	0.96[0.48-1.92]
DM2	1.41[0.24-8.34]	1.64[0.36-7.50]	1.52[0.34-6.79]
IGT and DM2	0.79[0.34-1.86]	1.07[0.54-2.12]	1.03[0.53-2.00]

Notes. WC, waist circumferences; IGT, Impaired Glucose Tolerance, DM2, Diabetes mellitus type 2

## DISCUSSION

It is well known that incidence of vitamin D deficiency increases in elderly people [1-4,27]. On the other hand, geographic location could play an important role in vitamin D status. St. Petersburg, a North-Western region of Russia, as well as most of other Russian regions, are located higher than 42° North latitude and has approximately 62 sunny days per year, a fact that predispose to sunlight and vitamin D deficiencies. Recent Russian studies showed that around 60% of children and adolescents in Moscow and 43% in St. Petersburg as well as more than half of the elderly population of Yekaterinburg had vitamin D deficiency [28,29]. However, our results revealed a high incidence of vitamin D insufficiency and deficiency in women of premenopausal and perimenopausal age regardless of the season. The possible factor that might contribute to the lack of 25(OH)D was the geographical location characterized by low level of sunlight. Our results confirmed a high prevalence of obesity, implicating calcidiol deficiency in overweight and obese people [16-18,30]. Previous studies showed that increased body fat is associated with lower 25(OH)D levels due to calcidiol accumulation in fat tissue, while our results demonstrated that low 25(OH)D level could predispose to fat accumulation.

The presence of VDR in adipocytes suggests that vitamin D plays a role in lipogenesis and lipolysis regulation [19,31]. It was shown that in vitro active form- 1,25(OH)_2_D - could regulate adipocyte death and decrease fat mass. On the other hand, a reduction in 25(OH)D concentration may lead to an increase in serum iPTH, that lead to regulation of body fat mass, increasing lipogenesis and decreasing lipolysis [32-34]. Our results demonstrated significant associations between iPTH level and fat mass index (r=0.34, p<0.05) that could confirm this theory.

Like obesity has become a global epidemic and a risk factor for diabetes type 2 [45-37], also the vitamin D endocrine system could be involved in glucose homeostasis and in insulin release mechanisms. Epidemiological studies suggested that vitamin D deficiency may increase the risk of developing insulin resistance and diabetes [38-40]. Our data may denote the regulatory role of 25(OH)D in the function of pancreatic β-cells and the level of insulin sensitivity. This proposition is supported by correlation between calcidiol levels and stimulated insulin levels, insulin resistance, ß-cells functional activity as well as insulin sensitivity of tissues. However, as shown here, vitamin D deficiency had the most pronounced effect either on insulin secretion by the pancreatic cells in people with normal weight, or, in the case of overweight or obesity, it lead to low tissue insulin sensitivity. Our results are supported by other studies [5,11,15,38,39]. Hence, the beneficial effect of vitamin D in glucose metabolism cannot be ignored as a potential preventive and even therapeutic measure for obesity and diabetes.

Finally, we would like to mention that the limited number and gender of study subjects, outpatient basis of the study precluding dynamic methods such as euglycemic clamp, and inability to perform accurate assessment of ergocalciferol consumption with food and cholecalciferol synthesized in the skin under the action of ultraviolet light might have affected the data. Future studies to evaluate the impact of 25(OH)D status on weight and glucose metabolism parameters in other populations, such as young people including men, are needed.

## MATERIALS AND METHODS

The 320 women who took part in the research ranged in age from 40 to 52 y.o. The exclusion criteria were: calcium or vitamin D therapy, long or frequent exposure to sunlight, as well as diabetes mellitus, significant liver or kidneys disease and malabsorbtion syndrome. The study was performed over the period from September to May. All women provided written consent.

Anthropometric examination included height and weight measurements with the use of a calibrated balance beam scale and a wall-mounted stadiometer; calculation of body mass index (BMI); and measurement of waist circumferences (WC) using standard methods. Normal weight was defined as BMI<25 kg/m^2^, overweight as 25≤BMI<30 kg/m^2^ and obesity as BMI≥30 kg/m^2^ [41]. Dual energy absorbtiometry (DEXA, Lunar Prodigy, USA) was performed for 134 women. Distribution (android, gynoid fat, trunk % fat) and amount (total fat) of fat mass was calculated automatically. The fat mass index (FMI) was calculated using fat mass measurement [42,43].

Fasting plasma glucose was determined enzymatically using commercially available kits and auto analyzer (UniCel DxC 800, USA). Serum insulin was measured using enzyme immunoassay kits (Beckman Coulter, USA). Homeostasis model assessment estimates of IR (HOMA-IR) and ß-cells function (HOMA-B) were calculated using fasting glucose and insulin measurements [44]. Standard 75-g oral glucose tolerance test was performed for 250 subjects. Insulin sensitivity index (ISI-(0,120)) was calculated using fasting and 120-min glucose and insulin measurements [45].

Serum 25(OH)D was measured using immunoassay kits (Immunodiagnostic System Ltd, UK) with quality control materials provided by the manufacturer. Status of vitamin D was classified as: normal - 25(OH)D levels higher than 75 nmol/L; insufficient - 50 to <75 nmol/L; and deficient - low than 50 nmol/L [2,8].

Serum intact parathyroid hormone level (iPTH) was detected using ELISA (Access) and commercial immunoassay kits (Beckman Coulter, USA).

The data below are represented as means ± standard error or percentage. Statistical processing of the data was performed using the STATISTICA program system for Windows (version 5.5). Comparison of frequency characteristics of qualitative indicators was done using nonparametric methods χ^2^. Comparison of quantity indicators was performed using ANOVA module. To find the correlation between the studied indicators we applied Pearson correlation analysis.
